# Free Dispensary

**Published:** 1867-10

**Authors:** 


### *Free Dispensary.*

Hereafter, there will be a daily *clinique* at the College throughout the year—Sundays and holidays not excepted. The attendants will be treated gratis, and in cases of necessity, medicines will be furnished free of charge. A large corps of clinical assistants has been organized, and in every department of practice of medicine, surgery, or obstetrics, (*including* the specialties,) it is intended that every want shall be supplied.

The grand advantages of this great city are to be fully improved. The field has, up to this time, been unoccupied, for Rush College of old had not room for the exercise of its powers. But now it has the *pou sto* and proposes to use the lever. Coöperation of friends both in city and country is solicited. The poor of the city will be treated at their residences, under the supervision of the Faculty, at all necessary hours, day or night.

